# Translocation junctions in *TCF3-PBX1* acute lymphoblastic leukemia/lymphoma cluster near transposable elements

**DOI:** 10.1186/1759-8753-4-22

**Published:** 2013-10-17

**Authors:** Nemanja Rodić, John G Zampella, Toby C Cornish, Sarah J Wheelan, Kathleen H Burns

**Affiliations:** 1 Department of Pathology, Johns Hopkins University, School of Medicine, 600 North Wolfe Street, Carnegie 401, Baltimore, MD 21205, USA; 2 Department of Dermatology, Johns Hopkins University, School of Medicine, Baltimore, MD 21205, USA; 3 Howard Hughes Medical Institute, 4000 Jones Bridge Road, Chevy Chase, MD 20815-6789, USA; 4 Department of Oncology, Division of Biostatistics and Bioinformatics, Johns Hopkins University, School of Medicine, Baltimore, MD 21205, USA; 5 McKusick-Nathans Institute of Genetic Medicine, 601 N Caroline St #5212, Baltimore, MD 21287, USA; 6 Sidney Kimmel Comprehensive Cancer Center, 401 N Broadway, Baltimore, MD 21287, USA; 7 High Throughput Biology Center, Johns Hopkins University School of Medicine, Baltimore, MD 21205, USA

## Abstract

**Background:**

Hematolymphoid neoplasms frequently harbor recurrent genetic abnormalities. Some of the most well recognized lesions are chromosomal translocations, and many of these are known to play pivotal roles in pathogenesis. In lymphoid malignancies, some translocations result from erroneous V(D)J-type events. However, other translocation junctions appear randomly positioned and their underlying mechanisms are not understood.

**Results:**

We tested the hypothesis that genomic repeats, including both simple tandem and interspersed repeats, are involved in chromosomal translocations arising in hematopoietic malignancies. Using a database of translocation junctions and RepeatMasker annotations of the reference genome assembly, we measured the proximity of translocation sites to their nearest repeat. We examined 1,174 translocation breakpoints from 10 classifications of hematolymphoid neoplasms. We measured significance using Student’s *t*-test, and we determined a false discovery rate using a random permutation statistics technique.

**Conclusions:**

Most translocations showed no propensity to involve genomic repeats. However, translocation junctions at the transcription factor 3 (*TCF3*)/E2A immunoglobulin enhancer binding factors E12/E47 (E2A) locus clustered within, or in proximity to, transposable element sequences. Nearly half of reported *TCF3* translocations involve a MER20 DNA transposon. Based on this observation, we propose this sequence is important for the oncogenesis of *TCF3-PBX1* acute lymphoblastic leukemia.

## Background

Genomic rearrangements can occur in germline nuclei, resulting in inherited diseases, or in somatic nuclei, contributing to tumorigenesis. The latter can vary from complex events such as chromothripsis, to relatively simple abnormalities such as recurrent chromosomal translocations; the underlying mechanisms remain unclear. Genomic rearrangements have been induced in mammalian cell cultures in few systems [1-3]. Although these *in vitro* generated translocations provide a valuable experimental tool, the engineered translocation partner sequences rarely match known oncogenic translocation sequences [4].

Most recognized genomic rearrangements in human cancers today are not resolved at the nucleotide level. Widely used assays include karyotyping, fluorescence in situ hybridizations, and microarray platforms with probes for comparative genomic hybridization and single nucleotide polymorphism genotyping. None provides nucleotide resolution of translocation breakpoints; massively parallel short-read sequencing has this ability, particularly when tailored approaches are used to 'rescue’ alignments of reads spanning the breakpoints. However, highly repetitive intervals at breakpoints may be a confounding factor.

Breakpoints resolved precisely can provide insights into the mechanisms responsible for rearrangements. For example, some hematolymphoid neoplasm breakpoints are marked by the presence of cryptic heptamer/nanomer sequences [5]. Similarly, Translin protein binding sequences have been detected near chromosomal breakpoints in lymphoid neoplasms [6]. In both scenarios, DNA sequence is a key participant in the mechanism of translocation.

We chose to look for evidence of genomic repeat involvement in chromosomal translocations that drive human hematopoietic malignancies. Repetitive sequences comprise nearly half of the human genome; many are interspersed repeats reflecting insertions of mobile DNA sequences [7]. Because of their prevalence in genomes, these repeats are intrinsic substrates for homologous recombination and single strand annealing reactions [8,9]. For unknown reasons, repeating elements are also disproportionately involved in non-homologous end joining events at specific loci. One example of this occurs in a mouse model of *MYC*-induced lymphoma, which shows increased LINE-1 retrotransposon sequences at break sites with no homology or short microhomologies (1–4 bp) suggestive of non-homologous end joining [10].

To address the question, we took advantage of two resources, the RepeatMasker annotation of the reference human genome assembly [http://www.repeatmasker.org], and a compilation of more than 1,000 chromosomal translocation spanning sequences curated by the Liber laboratory [11]. For each translocation junction, we measured distance to the nearest repeat. To avoid erroneous associations between translocation junctions and repeats, we compared randomly permuted positions within the translocation gene locus.

## Results and discussion

**Figure 1:**
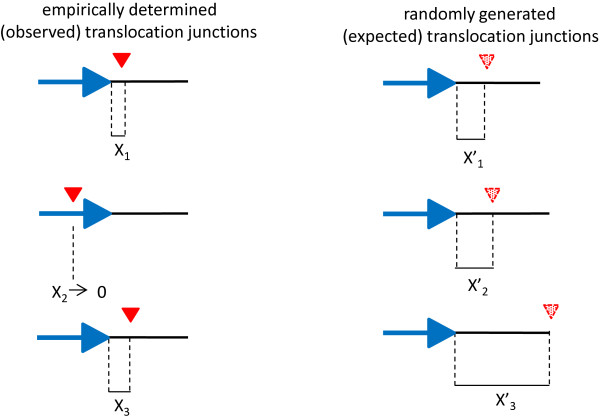
**Experimental outline depicting a hypothetical translocation region encompassing three translocation junctions.** An illustration on the left represents the hypothesis, where there is a spatial association (symbol X) between the three observed translocation junctions (red triangles) and the nearest repeated sequence (blue arrow). Similarly, an illustration on the right represents the null hypothesis, where there is no spatial association (symbol X’) between three randomly generated translocation junctions (broken triangles) and their nearest repeat (blue arrow). We compared actual translocation junctions to 1,000 randomly generated positions to identify translocation junction regions that consistently happen near repeats.

Translocation junctions from ten types of hematolymphoid neoplasm (Table 1) were analyzed to determine whether these occurred within or closer to the nearest repeat than would be expected by chance (Figure 1). The percent of translocation junctions occurring within repeat intervals varied, partly as a reflection of repeat content at the involved gene loci. For example, 67% of translocation junctions in both transcription factor 3/transcription factor E2-alpha (*TCF3*) and abelson murine leukemia viral oncogene homolog 1 (*ABL1*) were present in repeats (Table 2). In contrast, only 2–3% of junctions in runt-related transcription factor 1; translocated to, 1 (*RUNX1T1*) were in repeats (Table 2). The longest average and shortest average observed distances between translocations and their nearest repeat were 684 bp and 1 bp in T-cell receptor alpha chain (*TCRA*) and *TCF3*, respectively (Table 2).

**Table 1:** Translocation regions studied

**Genetic abnormality**	Clinical entity*	**Rearrangement**	**Junctions‡**
TCF3-PBX1	Pre-B/B-ALL	t(1;19)(q23;p13.3)	60
BCR-ABL1	CML	t(9;22)(q34;q11)	67
t-MLL	Therapy AML	t(9;11)(q22;q23)	26
MLL	Primary ALL and AML	t(4;11)(q21;q23)	424
		t(9;11)(q22;q23)	24
ETV6-RUNX1	Pre-B/B-ALL	t(12;21)(p12;q22)	105
RUNX1-RUNX1T1	AML	t(8;21)(q22;q22)	132
MYC-IGH	Sporadic BL	t(8;14)(q24;q32)	178
BCL6-IGH	Mature B lymphomas	t(3;14)(q27;q32)	52
SCL-TCRA	Pre-T/T ALL	t(1;14)(q32;q11)	48
LMO2-TCRA	Pre-T/T ALL	t(11;14)(q13;q11)	58
Total			1174

TCF3: transcription factor 3; PBX1: pre B-cell leukemia transcription factor 1; BCR: breakpoint cluster region; ABL1: Abelson murine leukemia viral oncogene homolog 1; MLL: myeloid/lymphoid or mixed lineage leukemia gene; ETV6: ets variant gene 6; RUNX1: runt-related transcription factor 1; RUNX1T1: runt-related transcription factor 1 translocated to, 1; MYC: v-myc avian myelocytomatosis viral oncogene homolog; IGH: IgG heavy chain locus; BCL6: B-cell lymphoma 6; SCL: stem cell leukemia hematopoietic transcription factor; TCRA: T-cell antigen receptor, alpha subunit; LMO2: lim domain only 2, t: therapy related.

*Distinct hematolymphoid neoplasms according to the World Health Organization classification; Pre-B/B-ALL: B lymphoblastic leukemia/lymphoma; CML: chronic myelogenous leukemia; Therapy AML: therapy-related acute myeloid leukemia; sporadic BL: Burkitt lymphoma; Pre-T/T-ALL: T lymphoblastic leukemia/lymphoma.

‡Number of translocation junctions examined.

**Table 2:** Repeat features at the translocation regions studied

**Translocation junction regions**	**Junctions occurring in repeats (%)**	**Junction to the nearest repeat‡ (bp)**		***P *** **value for interaction**
**E2A = PBX1 (TCF3-PBX1)**		**Observed**	**Expected**	
TCF3	20 (67)	1	42.46	<0.001†/<0.001*
PBX1	1 (3)	384.2	272.24	0.999
BCR-ABL				
BCR	14 (35)	293.5	251.37	0.978
ABL	18 (67)	37.74	171.48	0.017†/0.449*
Therapy-related MLLs				
t-MLL	2 (15.4)	34.54	95.38	0.373
t-MLLT3	3 (23)	242.85	264.64	0.949
MLLs rearranged				
MLL	72 (35.3)	156	108.78	0.999
AFF1	46 (21.1)	208.31	144.39	0.999
MLL	6 (50)	50.34	82.35	0.798
MLLT3	2 (16.7)	209.1	289.9	0.831
ETV6-RUNX1				
ETV6	15 (29)	121.11	113.64	0.987
RUNX1	6 (11)	284.54	245.3	0.966
RUNX1-RUNX1TL				
RUNX1	18 (27)	236.48	243.63	0.949
RUNX1TL	3 (2)	494.21	264.41	0.999
MYC-IGH				
MYC	8 (6)	399.1	116.44	0.999
IGH	29 (63)	668.86	298.77	0.999
BCL6-IGH				
BCL6	2 (5)	151.81	174.16	0.763
IGH	9 (64)	124.86	190.5	0.978
SCL-TCRA				
SCL	1 (6)	284.43	192.11	0.998
TCRA	5 (16)	648.96	396.16	0.999
LMO2-TCRA				
LMO2	5 (17)	160.86	208.56	0.704
TCRA	3 (10)	545.1	194.47	1

‡Distance, expressed in number of nucleotides, from translocation junction to the nearest repeat.

†*P* value for interaction between translocation junction to the nearest repeat (including repeating elements and tandem repeats).

*P value for interaction between translocation junction to the nearest repeating element.

**Figure 2:**
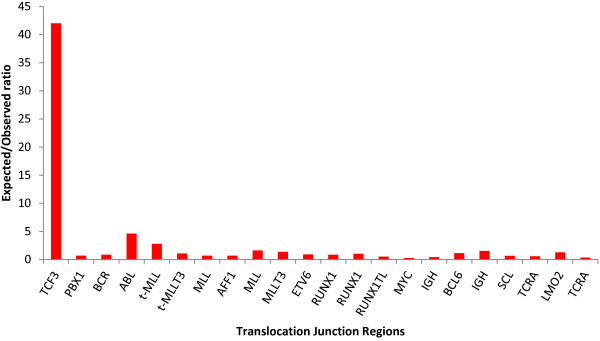
**Translocation junctions in *TCF3 *occur at or near repeats.** The Y-axis denotes the expected versus observed ratio of distances between translocation junctions and their nearest repeats. The X-axis denotes translocation loci analyzed. Other translocations examined were independent of local repeat content; expected versus observed ratios for these loci approach one (1). See Table 1 for abbreviations.

Next, we calculated ratios of the expected versus observed translocation-to-repeat distances (Figure 2). The largest ratio, reflecting a relative enrichment of translocation junctions in the vicinity of repeats, occurred in the *TCF3* translocation junction region (*TCF* locus ratio = 42, average ratio for other loci = 1.15) (Figure 2). Applying permutation based statistics, as described in the Methods section, confirmed significance of the enrichment of *TCF3* translocation junction at genomic repeats (n = 30; *P* <0.001) (Table 2). Using the same approach, we note a weaker association between translocations and genomic repeats at the *ABL1* region (n = 27; *P* = 0.017) (Table 2).

**Figure 3:**
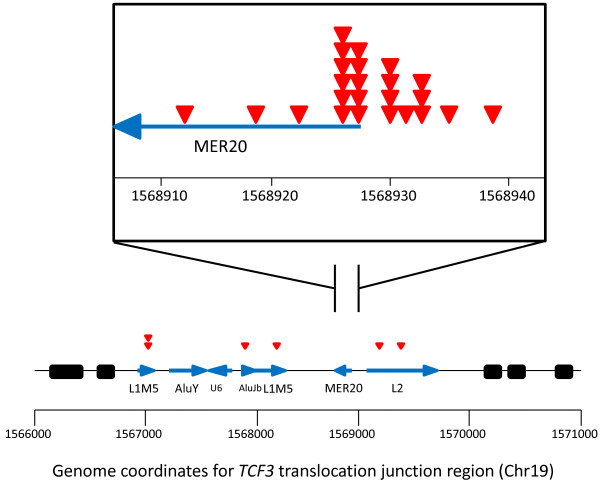
**Schematic representation of a *TCF3 *locus including translocations and transposable elements.** The red triangles represent individual translocation junctions, the blue arrows indicate transposable elements within TCF3, and the black rectangles identify TCF3 exons. Inset, TCF3 translocation junction density map within the MER20 transposon. Genome coordinates correspond to March 2006, NCBI36/hg18 human genome assembly. TCF3: Transcription factor 3; MER20: Medium reiteration frequency repetitive 20; L1: LINE-1 Long INterspersed Element 1; L2: Long INterspersed Element 2; Alu: Alu SINE; U6: Small nuclear RNA.

The TCF3 translocation junction region encompasses interspersed repeats from three categories, including a small nuclear RNA sequence (U6 snRNA), five retrotransposons, and a hAT-Charlie family DNA transposon (MER20). The retrotransposons at the locus include two Short INterspersed Elements (SINE) elements (AluY and AluJb), and three Long INterspersed Elements (LINE) elements (two L1M5s and a L2) (Figure 3). Interestingly, 14/30 (47%) of reported *TCF3* translocation junctions reside in the MER20 transposon (Figure 3); the distribution of MER20 embedded translocation junctions was non-random (Figure 3, inset).

Recurrent pathologic translocations occur in a wide range of human malignancies, from hematolymphoid cancers to carcinomas and sarcomas. As the genetics of these diseases are better characterized, specific lesions are being related to clinicopathological entities or even incorporated in their definition [12]. Sequence features at breakpoints can lend insights into how these events occur, and so we decided to investigate the prevalence of breakpoints with respect to genomic repeats. There have been other reports of non-uniform distributions of transposable element sequences at sites of chromosomal breaks. For example, nucleotide junctions demarking the postnatal chromosome 12p deletions in *ETV6-RUNX1* leukemia often occur at, or near, retrotransposon sequences [13].

In our study, we looked at rearrangement sites at 20 gene loci. Only *TCF*3 translocation sites exhibited clustering at or near transposable element sequences. All other translocation junctions from malignant proliferations of lymphoid and myeloid lineages showed random distributions relative to nearby repeats.

Our study leaves the mechanism unaddressed. How could *TCF3* repeats create a site susceptible to breakage or otherwise involve the locus in events leading to the translocation? It is possible that very short sequences also occurring randomly are sufficient. Prior work by Tsai et al. has shown that dsDNA breaks at the *TCF3/E2*A locus leading to translocations occurring in clusters at CpG dinucleotides [11]. This is similar to some other hotspots for breaks occurring the pro-B/pre-B stage of B-cell maturation. Of note, though, CpG nucleotides are not at break sites seen in the TCF3 fusion partner locus, pre-B-cell leukemia homeobox 1 (PBX1). CpG dinucleotides occurred on 53% of *TCF3* translocation junctions, while transposable elements were found on 67% of *TCF3* translocation sites.

It is also possible that a lengthier protein recognition sequence is important near the break site. Transposable elements can contain, for example, transcription factor binding sites and other regulatory protein binding sites important for transcriptional control around the repeat [14,15]. Indeed, MER20 DNA transposons provide *cis*-regulatory sequences critical for inducing the transcription of prolactin during pregnancy and have been implicated in endometrial gene recruitment in the evolution of placental mammals [14,16,17].

## Conclusions

In summary, we analyzed 1,174 translocation sequences from ten hematolymphoid neoplasms for proximity to nearby repeats. Of these, *TCF3* translocation junctions were seen to cluster at or near transposable elements in a majority of *TCF3-PBX1* acute lymphoblastic leukemia. It is possible that the involved transposable element sequences are inherently susceptible to dsDNA breaks. Further studies will be needed to address sequence requirements for *TCF3-PBX1* and other leukemogenic translocations.

## Methods

### Translocation junction sequences

Genomic DNA from human clinical samples was extracted and translocations were Sanger sequenced by numerous independent investigators [11]. Published sequences assembled by Tsai et al. are publically accessible in a repository, herein referred to as the Lieber database (http://lieber.usc.edu/Data.aspx) [11]. The Lieber database includes translocation junction sequences, translocation genomic coordinates (hg18), and limited clinical data from various hematolymphoid neoplasms that are associated with recurrent translocations. We downloaded this information (Table 1), and analyzed loci with ten or more translocation breakpoints (Additional file 1).

### Mapping breakpoints with respect to repeats

Distances between each translocation junction and its nearest repeat element were determined by a Perl script (Additional file 2). Briefly, each translocation junction was aligned to its corresponding sequence in the March 2006 GRCh36/hg18 assembly version of the human genome. Translocation was annotated for repetitive sequences using Tandem Repeat Finder and RepeatMasker. We included the two major categories of genomic repeats: tandem repeats and interspersed repeats. The number of nucleotides between the translocation and its nearest repeat were then calculated, considering upstream and downstream sequences. For each locus, the observed distribution of distances was compared to distances found using random positions as substitutes for translocation junction (Figure 1).

### Statistical methods

For each of the twenty translocation intervals analyzed, we compared actual measurements between translocation junction and their nearest genomic repeats against the distances separating 1,000 random positions and their corresponding repeats. For each permutation, we calculated a Student’s *t*-value and its *P* value. For each of the twenty translocation intervals analyzed, we compared actual measurements between translocation junction and their nearest genomic repeats to the distances separating 1,000 random positions and their corresponding nearest repeats. Each translocation was compared to the distribution of distances created by the random sites using a one-sided Student’s *t*-test, to generate a *P* value; low *P* values indicate that the translocation is significantly closer to a repeat element than expected by random chance.

## Abbreviations

ABL1: Abelson murine leukemia viral oncogene homolog 1; Alu: (*Arthrobacter luteus*) element; BCL6: B-cell lymphoma 6; BCR: Breakpoint cluster region; E2A: Immunoglobulin enhancer binding factors E12/E47; ETV6: ets variant gene 6; FGF8: Fibroblast growth factor 8; IGH: IgG heavy chain locus; LINE-1: Long INterspersed element 1; LINE-2: Long INterspersed element 2; LMO2: LIM domain only 2; MER20: Medium reiteration frequency repetitive 20 element; MLL: Myeloid/lymphoid or mixed lineage leukemia gene; MYC: v-myc avian myelocytomatosis viral oncogene homolog; PBX1: Pre B-Cell leukemia transcription factor; RUNX1: Runt-related transcription factor 1; RUNX1T1: Runt-related transcription factor 1, translocated to, 1; SCL: Stem cell leukemia hematopoietic transcription factor; SINE: Short INterspersed element; snRNA: Small nuclear RNA; TCRA: T-cell antigen receptor, alpha subunit; TCF3: Transcription factor 3.

## Competing interests

The authors declare that they have no competing interests.

## Authors’ contributions

NR conceived of the study. NR, JZ, TCC, and KHB wrote the code to produce genomic distances. NR, SJW, and KHB performed statistical analyses. NR, SJW, and KHB drafted the manuscript. All authors read and approved the final manuscript.

## Supplementary Material

Additional file 1 **Nucleotide positions of translocation junctions examined.** Column A depicts a gene symbol that specifies one of the two translocation partners within a given hematolymphoid neoplasm with recurrent genetic abnormality. Column B denotes sequence used to determine translocation junction. Columns C and D denote chromosomal position and nucleotide position of translocation junction, relative to March 2006 Human Genome Assembly (hg18). Click here for file

Additional file 2 Program used to calculate translocation junction to repeat distance and to generate 1,000 random positions for each translocation region. Click here for file
